# Neuropharmacology of Cevimeline and Muscarinic Drugs—Focus on Cognition and Neurodegeneration

**DOI:** 10.3390/ijms22168908

**Published:** 2021-08-18

**Authors:** Patrik Oleksak, Michal Novotny, Jiri Patocka, Eugenie Nepovimova, Jakub Hort, Jan Pavlik, Blanka Klimova, Martin Valis, Kamil Kuca

**Affiliations:** 1 Department of Chemistry, Faculty of Science, University of Hradec Kralove, 50003 Hradec Kralove, Czech Republic; patrik.oleksak@uhk.cz (P.O.); eugenie.nepovimova@uhk.cz (E.N.); 2 Department of Neurology, Faculty of Medicine and University Hospital Hradec Kralove, Charles University, 50003 Hradec Kralove, Czech Republic; m.novas82@gmail.com (M.N.); blanka.klimova@uhk.cz (B.K.); martin.valis@fnhk.cz (M.V.); 3 Institute of Radiology, Toxicology and Civil Protection, Faculty of Health and Social Studies, University of South Bohemia in Ceske Budejovice, 37005 Ceske Budejovice, Czech Republic; toxicology@toxicology.cz; 4 Biomedical Research Centre, University Hospital, 50003 Hradec Kralove, Czech Republic; 5 Memory Clinic, Department of Neurology, 2nd Faculty of Medicine and Motol University Hospital, Charles University, 15006 Prague, Czech Republic; Jakub.hort@gmail.com; 6 Agora Pharmaceuticals, s.r.o., 50009 Hradec Kralove, Czech Republic; jan.pavlik@agora-pharmaceuticals.com

**Keywords:** cevimeline, evoxac, AF102B, SNI2011, cholinergic agonist, muscarinic receptors, cognition, memory, neurodegeneration, Alzheimer disease

## Abstract

At present, Alzheimer’s disease (AD) and related dementias cannot be cured. Therefore, scientists all over the world are trying to find a new approach to prolong an active life of patients with initial dementia. Both pharmacological and non-pharmacological pathways are investigated to improve the key symptom of the disease, memory loss. In this respect, influencing the neuromodulator acetylcholine via muscarinic receptors, such as cevimeline, might be one of the therapeutic alternatives. The purpose of this study is to explore the potential of cevimeline on the cognitive functions of AD patients. The methodology is based on a systematic literature review of available studies found in Web of Science, PubMed, Springer, and Scopus on the research topic. The findings indicate that cevimeline has shown an improvement in experimentally induced cognitive deficits in animal models. Furthermore, it has demonstrated to positively influence tau pathology and reduce the levels of amyloid-β (Aβ) peptide in the cerebral spinal fluid of Alzheimer’s patients. Although this drug has not been approved by the FDA for its use among AD patients and there is a lack of clinical studies confirming and extending this finding, cevimeline might represent a breakthrough in the treatment of AD.

## 1. Introduction

Alzheimer’s disease (AD) is an irreversible and progressive neurodegenerative disorder that affects memory and thinking skills, and eventually, leads to the inability to execute the activities of daily living [1,2]. In fact, it is the fifth leading cause of death in the world [3] and the most common form of dementia, accounting for 60–70% of all dementias [4]. The main symptom of AD and other dementias is memory loss. In the early stages of AD, especially short-term memory, i.e., a brief period of time in which one can recall information that s/he was just exposed to, for example a phone number or a meal s/he has just eaten, is affected [5]. However, in the advanced stages of the disease, patients have problems with their long-term memory, i.e., the memory that refers to the storage of information over an extended period [5]. This type of memory includes three different types of memories, all of which are significantly impaired in later stages of AD: semantic memory associated with understanding the meaning of words, episodic referring to the past events, and procedural associated with an ability to carry out a task.

Currently, 44 million people worldwide are living with AD or a related form of dementia, out of which 10 million are people in Europe [6]. By 2030, this number should rise to 14 million, which would inevitably burden national health systems, state economies, and the daily lives of millions of people with AD and their families [7]. The expected costs of treating these people are estimated at €250 billion. Although AD and related dementias cannot be cured, their progression can be delayed. Therefore, scientists all over the world are trying to find a new approach to prolong an active life of patients with initial dementia. Both pharmacological and non-pharmacological pathways are investigated to improve the key symptom of the disease, the memory loss. In this respect, imitating the neurotransmitter acetylcholine via acting on the muscarinic receptors might be one of the therapeutic alternatives based on the available literature. 

Acetylcholine (ACh) is a neurotransmitter of special importance. It can be found in the peripheral and central nervous system (CNS). In the CNS, it plays an important role in influencing alertness, attention, learning, and memory. Damage to this cholinergic system has been found to be closely associated with decreased cognitive function and AD manifestations. There are two groups of ACh receptors: nicotinic and muscarinic. The cholinergic system can be therapeutically affected by either direct-acting or indirect-acting cholinergic agents. The second group of cholinergics, reversible substances, plays an important role in the treatment of AD. This group includes rivastigmine, donepezil, and galantamine [8].

**Figure 1:**
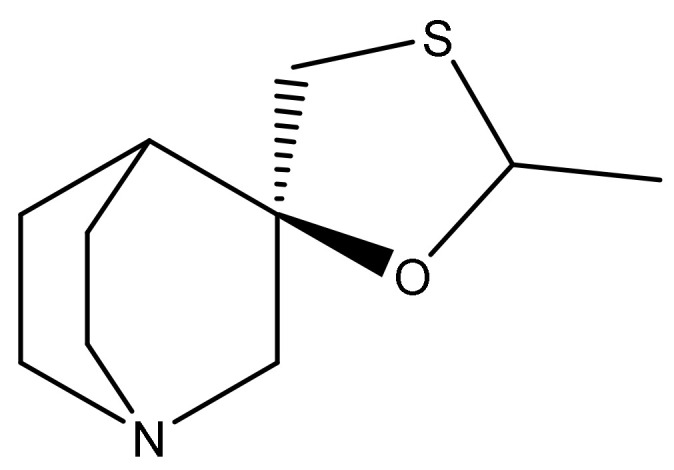
The structure of cevimeline.

Cevimeline (Figure 1) represents a potent direct-acting muscarinic receptor agonist. Chemically, it is 2-methylspiro[1,3-oxathiolane-5,3′-1-azabicyclo[2.2.2]octane]. In the literature or clinical studies, it is also known as AF102B, FKS-508, SNK-508, SND-5008, or SNI-201. Its oral dosage form has been registered by the FDA under the tradename Evoxac^®^ in indication “treatment of symptoms of dry mouth in patients with Sjögren’s Syndrome”, and the recommended dose of cevimeline hydrochloride is 30 mg taken three times a day. The major mechanism of action of this muscarinic agonist (in sufficient dosage) is increased secretion of exocrine glands, such as salivary and sweat glands and increased tone of the smooth muscle in the gastrointestinal and urinary tracts [9,10]. Cevimeline was also studied as a treatment for dry mouth caused by radiation therapy to the head and neck (NCT00466388; NCT00017511) [8]. There is currently no (according to clinicaltrials.gov) clinical trial with cevimeline. However, extensive experimental preclinical research indicates its ability to positively affect cognitive function and shows its potential in the treatment of AD. 

Therefore, the purpose of this study is to explore the potential of cevimeline on cognitive functions of AD patients.

## 2. Results and Discussion

### 2.1. Pharmacology of Cevimeline

Cevimeline hydrochloride is a white crystalline powder that is soluble in water [9]. Absorption: After peroral administration of a single dose (30 mg), cevimeline is rapidly absorbed with a mean time to peak concentration of 1.5 to 2 h. When administered with food, there is a decrease in the rate of absorption, with a fasting T_max_ of 1.53 h and a T_max_ of 2.86 h after the meal [9]. In healthy volunteers using cevimeline (30 mg, t.i.d, 7 d), C_max_ was 59.9 ng/mL and it was reached in 1.8 h. In a group of healthy elderly volunteers (aged 62–80 years; 30 mg), a mean C_max_ of 90.8 ng/mL was reached on average over 1.5 h. In women with Sjögren’s syndrome who were given cevimeline (30 mg), a mean C_max_ of 91.6 ng/mL was reached in an average of 1.5 h [11]. No accumulation of active drug or its metabolites was observed, following the multiple dose administration [12]. Distribution: An approximate distribution volume of cevimeine is 6 L/kg, and its binding to human plasma proteins is less than 20%. Therefore, extensively, cevimeline’s binding to tissues is suggested, but the specific binding sites remain unknown. Metabolism: Cevimeline is metabolized by isozymes CYP2D6, CYP3A3, and CYP3A4. Within the first 24 h, 86.7% of the administered dose was recovered as follows: 16.0% uncharged, 44.5% *cis*- and *trans*-sulfoxide, 22.3% glucuronic acid conjugate, and 4% cevimeline *N*-oxide. *Trans*-sulfoxide, as the metabolite of cevimeline, was converted into the corresponding conjugate with glucuronic acid in approximately 8%. No inhibition of cytochrome P450 isozymes 1A2, 2A6, 2C9, 2C19, 2D6, 2E1, and 3A4 was observed for cevimeline [9]. Excretion: Cevimeline’s mean half-life is 5 ± 1 h. The administration of a 30 mg dose of cevimeline reveraled that 84% of the dose was excreted in the urine after 24 h. Moreover, 97% of the administered dose was recoverd in the urine, whereas only 0.5% of the dose was recovered in the feces after seven days [9,13,14,15]. Mechanism of action: Cevimeline is a cholinergic agonist that binds and activates M1 and M3 muscarinic receptors. The selectivity of cevimeline toward mAChR was evaluated by Heinrich et al. [16]. The EC_50_ values for cevimeline were determined in the study as follows: 0.023 μM for M1, 1.04 μM for M2, 0.048 μM for M3, 1.31 μM for M4, and 0.063 μM for M5 receptors. This confirmed that cevimeline is a potent M1 agonist. As seen from the data described above, its selectivity toward M3 was 2-fold lower and toward M5 was 3-fold lower if compared with the selectivity for the M1 receptor. However, the selectivity of cevimeline toward the M2 receptor was 46-fold lower and toward the M4 receptor was 43-fold lower than its selectivity for the M1 receptor [16]. It should be mentioned that M5 receptors are expressed in the regions discrete of therapeutic interest. The M5AChR subtype represents in total mAChR population in the CNS less than 2% [17]. M1 receptors are primarily found in the secretory glands (exocrine glands, such as the salivary and sweat glands). The activation of the M1 receptors leads to the increased secretion from the secretory glands. In general, M3 receptors are distributed on the smooth muscle and in many glands that help stimulate secretion in the salivary glands. The activation of M3 receptors leads to the contractions of the smooth muscle and to the increased glandular secretion. As a result, the increased salivary excretion is observed, and dry mouth symptoms are relieved [18]. That is why cevimeline was previously approved for the treatment of xerostomia in Sjögren’s syndrome. Adverse effects: The toxicity of cevimeline is defined by an exaggeration of its parasympathomimetic effects (visual disturbance, lacrimation, gastrointestinal spasm, nausea, vomiting, diarrhoea, headache, sweating, or respiratory distress) [9]. Voskoboynik et al. reported a previously healthy patient who deliberately ingested approximately 10 mg/kg of cevimeline and showed symptoms of muscarinic excess and mental depression. This incident is in detail characterized below [19].

### 2.2. Muscarinic Acetylcholine Receptors and Cognition

**Figure 2:**
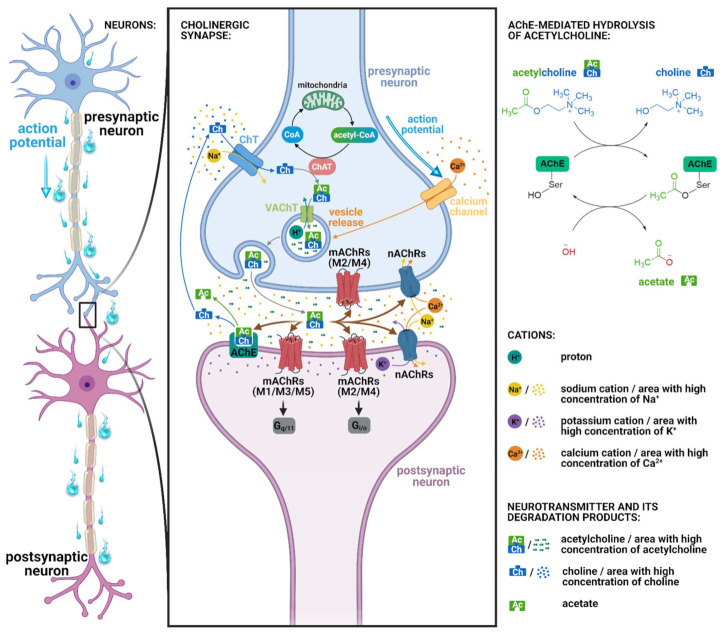
Left: Connection of presynaptic and postsynaptic neuron. Middle: Simplified illustration of cholinergic synapse with the mode of action of acetylcholine. The influx of Ca^2+^ into the presynaptic neuron caused by action potential is mediated via calcium channels and results in synaptic vesicle fusion to the active zone membrane of the presynaptic neuron. The subsequent acetylcholine release into the synaptic cleft enables the activation of acetylcholine receptors on the postsynaptic membrane. The acetylcholine breakdown mediated by acetylcholinesterase generates acetate and choline. Molecules of choline are reabsorbed by presynaptic neuron via choline transporters. Choline acetyltransferase-mediated acetylation of choline regenerates acetylcholine. Then, the neurotransmitter is transported into the synaptic vesicle and stored until the next use. Top-right: Simplified scheme of acetylcholine hydrolysis by acetylcholinesterase with subsequent enzyme regeneration. Bottom-right: Legend of involved cations, neurotransmitter, and its degradation products. Abbreviations: Ac, acetate (or acetyl group if combined with choline); AChE, acetylcholinesterase; Ch, choline; ChAT, choline acetyltransferase; ChT, choline transporter; CoA, coenzyme A; G_i/o_ and G_q/11_, G proteins; mAChRs, muscarinic acetylcholine receptors; nAChRs, nicotinic acetylcholine receptors; VAChT, vesicular acetylcholine transporter. Created with BioRender.com.

**Figure 3:**
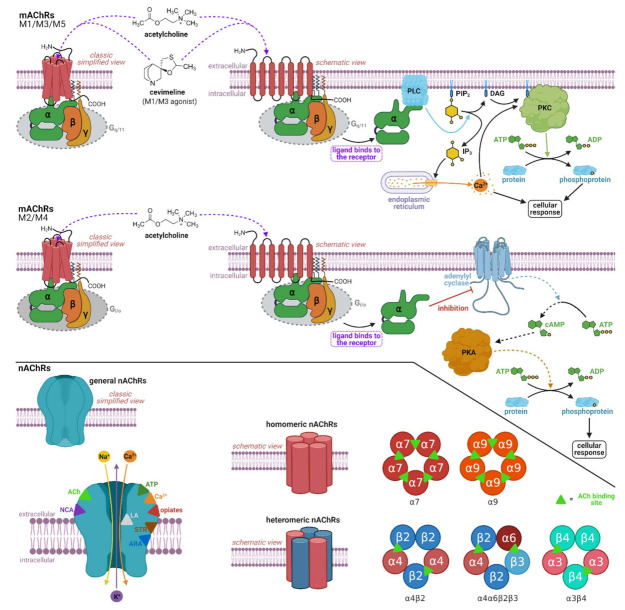
Top: Simplified and schematic view of muscarinic acetylcholine receptors (mAChRs) type M1, M3, and M5 and their signaling pathway. The receptors are coupled to trimeric G_q/11_ proteins composed of α-, β-, and γ-subunits. Upon ligand binding, mAChR undergoes a conformational change, resulting in dissociation of the α-subunit, which activates phospholipase C. Activated phospholipase C promotes the hydrolysis of phosphoinositol 4,5-bisphosphate. One of the hydrolysis products, diacylglycerol, promotes the translocation of protein kinase C from the cytoplasm to the cell membrane followed by the activation of kinase. Activated kinase mediates the phosphorylation of various proteins, which activate several gene transcription factors. The second product of the hydrolysis, inositiol 1,4,5-trisphosphate, is translocated to its receptors integrated into endoplasmic reticulum, resulting in the opening of calcium channels. Increasing intracellular calcium promotes several cellular responses. Middle: Simplified and schematic view of mAChRs type M2 and M4 including signaling pathway of the receptors coupled with trimeric G_i/o_ proteins. Upon ligand binding to the binding site of the receptor, the dissociated α-subunit inhibits adenylyl cyclase. The inhibition of adenylyl cyclase results in a decreased level of intramolecular cAMP. Reduced levels of cAMP as a second messenger decreases an activity of cAMP-dependent protein kinase A, which catalyzes the phosphorylation of several proteins followed by various cellular responses. Bottom: Simplified view of nicotinic acetylcholine receptors (nAChRs). The receptors are composed of five subunits and act as natural ion channels. Channels allow Na^+^, Ca^2+^ and K^+^ ions to flow down their electrochemical gradient; some of the channels enable the flow of selected cations only. The function of nAChRs can be modulated by neurotransmitters as well as by different toxins and drugs. The cross-section of the nAChR indicates binding sites of various modulators (colored triangles) and cation flow across the pore (colored arrows). The pentameric schematic view of nAChRs illustrates two main groups of the receptors (homomeric and heteromeric) with selected examples (α7, α9, α4β2, α4α6β2β3, and α3β4). The acetylcholine binding sites on nAChRs are depicted by green triangles. Abbreviations: ACh, acetylcholine; ADP, adenosine diphosphate; ARA, arachidonic acid; ATP, adenosine triphosphate; cAMP, cyclic adenosine monophosphate; DAG, diacylglycerol; G_i/o_ and G_q/11_, G proteins; IP_3_, inositiol trisphosphate; LA, local anesthetics; NCA, noncompetitive agonists; PIP_2_, phosphatidylinositol 4,5-bisphosphate; PLC, phospholipase C; PKA, protein kinase A; PKC, protein kinase C; STR, steroids. Created with BioRender.com.

Acetylcholine is a major neurotransmitter in the body. Receptors for this neurotransmitter are of two types: acetylcholine muscarinic and acetylcholine nicotinic (Figure 2). There are five subtypes of muscarinic receptors known as M1, M2, M3, M4, and M5 [20], and classically, there have been two subtypes of nicotinic receptors: NM (muscle-type; stimulation muscle contraction; increase Na^+^ and K^+^ permeability) and NN (neuronal-type; stimulate all autonomic ganglia; Na^+^, K^+^, and Ca^2+^ channel opening), although recently, they have been found also in the non-neuronal tissues [21,22]. Nicotinic acetylcholine receptors (nAChRs) are ligand-gated ion channels [23]. The nAChR consists of five subunits and is assembled into homomeric or heteromeric pentamers. In mammals including humans, 16 subunits that are designated by Greek letters often supplemented with Arabic numerals (α1–α7, α9–α10, β1–β4, γ, δ, ε), have been identified. The α8 subunit is related to the α7 subunit, but it has been identified only in avian species [24,25,26]. Muscarinic acetylcholine receptors (mAChRs) have seven transmembrane regions. They are known as G protein-coupled receptors; therefore, they transduce their signals through G proteins. Subtypes M1/M3/M5 are coupled with G_q/11_ protein, while M2/M4 subtypes are coupled with G_i/o_ protein [17]. Furthermore, mAChRs M1, M3, and M5 are combined to stimulate phospholipase C, while mAChRs receptors M2 and M4 act to inhibit adenylyl cyclase (Figure 3). mAChRs are present in both the periphery and the CNS. mAChRs in the central and peripheral nervous system are involved in autonomic, cognitive, and motor function. M1AChRs are the most common subtype in the CNS and are abundant in the postsynaptic nerve endings of all areas of the forebrain [27,28]. Many studies suggest that M1AChRs are critically involved in cognitive processes and that loss of cholinergic function contributes to the cognitive decline associated with AD and other neurological and psychiatric disorders [29,30,31]. Neurodegenerative disorders are associated with impairment of a number of cognitive domains and deterioration of episodic memory, which underlies significant difficulties for patients in daily activities, including self-care [32]. Damage to muscarinic M1 receptors and their associated signaling pathways plays an important role in these disorders [33,34]. Therefore, pharmacological modulation of muscarinic M1 receptors offers the possibility of improving episodic memory [35,36].

Acetylcholine plays an important role in cognitive effects that are mediated by stimulation of the M1 receptors. Therefore, M1 receptor agnosists may improve cognition upon their interaction with the receptors. This improvement has been achieved in animal models as well as in cognitive tests of patients suffering from AD. In addition, central presynaptic M2 receptor antagonists may also improve cognition upon increasing acetylcholine release. Acetylcholine M1 receptor agonists have been shown to increase the activity of medial prefrontal cortical neurons, restoring reverse learning disabilities [37]. Such specific M1 agonists can be used to improve memory by enhancing cholinergic communication in the brain [38]. M1 muscarinic agonists may modulate some of the hallmarks of AD, improving its course, with important implications for future therapy. Tsang et al. showed that the change in neuropsychiatric behavior in AD patients is due to a drastic decrease in M2 receptors [39]. Earlier studies reported by Zuchner et al. suggested that stimulation of the M2 receptor is associated with a decrease in β-secretase enzyme activity [40,41]. Thus, a decrease in the M2 receptor is thought to lead to an increase in β-amyloid peptides in the brain [42]. It has also been reported that α-secretase activity, and thus less β-amyloid peptide production, increases after stimulation of M1/M3 receptors [43].

The possible involvement of muscarinic acetylcholine receptors in various pathological conditions is becoming an important area of neuropharmacology. The treatment of various pathological conditions based on muscarinic receptors as targets has long been limited by the lack of selective muscarinic receptor ligands. Today, some of such muscarinic receptor ligands are known as, for example, xanomeline [44], sabcomeline [45], talsacidine [46], NGX-267 (AF267B) [47], WAY-132983 [48], and cevimeline [35] as well. These M1 agonists are classified as orthosteric ligands. Although the majority of the orthosteric ligands failed in clinical development at some stage probably due to reduced selectivity toward M1 receptors, they provided interesting data about the therapeutic potential of M1 agonists. Moreover, some of the compounds entered phase II/III of clinical studies [16]. Bodick et al. reported a placebo-controlled clinical trial of xanomeline, which is a direct acting muscarinic receptor with a possible therapeutic effect in AD patients. The highest administered dose of xanomeline tartrate seemed to improve a cognitive performance in patients that completed the study. Unfortunately, dose-dependent adverse effects (mainly gastrointestinal) resulted in the limitation of the drug utility. The authors remarked that xanomeline-mediated cognitive improvement seemed to be equally in magnitude to the improvement in cognition achieved in a tacrine hydrochloride-based study in AD patients [49,50]. However, the authors further claimed that these congnitive improvements were different in kind; therefore, additional studies are required. Moreover, the study also confirmed an improvement of behavioral disturbances in AD patients [49]. Interestingly, xanomeline, the M1/M4 receptor agonist, has been attracting attention in recent years due to its efficiency to treat behavioral and psychotic disturbances, as showed in the trials in AD patients. This resulted in further studies to evaluate its utility for the treatment of schizophrenia [51]. Indeed, xanomeline revealed its potential for a novel treatment of schizophrenia with improved short-term memory and verbal learning function [52]. Despite its gastrointestinal adverse effects, xanomeline still remains an interesting drug for further studies [53]. According to the above-mentioned studies, targeting mAChRs can be considered as an interesting approach for both cognitive and psychotic disturbances therapies [51].

In addition to the direct muscarinic receptor agonists, the allosteric modulators represent an alternative approach in the improvement of cognition. One of the most highlighted attributes in allosteric modulators is their selectivity toward M1 receptors. Threfore, it is assumed that the adverse effects as a result of nonselective activation of other mAChRs (M2-M5) should be reduced. There is a study reported by Chambon et al., which focused on the mode of action of benzyl quinolone carboxylic acid (BQCA), the M1 receptor positive allosteric modulator in relation to positive effects on memory and side effects in an animal model (rats). The findings of this study reveal that the therapeutic potential of BQCA for the treatment of memory deficits was observed in Alzheimer’s disease and that the results of this molecule proved an advantage over the agonist cevimeline [38]. In another study, Gould et al. confirmed a significant role of M1AChR in learning and memory of mice. The study revealed that the M1AChR knockout (M1KO) mice showed a reduced rate of learning in comparison to the wild-type mice. Moreover, the administration of the M1 positive allosteric modulator, BQCA, enhanced learning rate in the wild type, but it did not affect the M1KO in the discrimination task over 12 consecutive days. The authors further reported that M1AChR represented a significant potential therapeutic target for the improvement of cognition in neuropsychiatric disorders, such as schizophrenia [54]. Another animal study based on the other selective M1 muscarinic receptor positive allosteric modulator, PQCA, was evaluated by Uslaner et al. The study for the first time demonstrated improvements in cognitive performance in the rodent, cynomolgus macaque, and rhesus macaque. The authors concluded that the M1 selective modulator seems to be a suitable candidate for the trials in AD patients [55]. For the sake of completeness, it should be mentioned that a promising highly selective, positive allosteric modulator of the M1AChR, MK-7622, which entered the phase II trials in AD patients [56], has been recognized recently as ineffective at improving the cognition. The study in mild-to-moderate AD patients treated with MK-7622 did not confirm an expected improvement in cognitive functions. As declared by the authors, their findings based on MK-7622 trials do not necessary mean that other selective M1 allosteric modulators would be unable to improve cognition [57].

To conclude, the selective M1 allosteric modulators and direct-acting mAChR agonists are both considered as possible drugs to improve cognitive performance as well to treat the AD. Undoubtedly, both groups of compounds may provide significantly different biological data. However, the studies mentioned above and those discussed below demonstrate that mAChRs are still an attractive target associated with cognition and AD. Even in the case if the drugs will not phenomenally treat cognitive function, some of them still bear potential to attenuate progression of the AD. This can bring an improvement in the quality of life for AD patients and their families.

### 2.3. Cevimeline and Cognition

A relatively well-documented area is the effect of cevimeline on cognitive function in animal models (mice, rats, monkeys). Between 1988 and 2014, several experimental studies evaluating the effect of this drug on various forms of memory (reference memory, working memory, fear memory) on amnesia and learning-related processes were published [58,59,60,61,62,63,64,65,66,67]. Likewise, all authors conclude that based on these results, cevimeline may be considered as a candidate for the clinical examination of the cholinergic hypothesis of cognitive decline in Alzheimer’s dementia. 

Nakahara et al. explored the impact of cevimeline on experimental amnesia (produced by anticholinergic agents intracerebroventricularly and subcutaneously) using a passive avoidance task in rats. It was found that cevimeline is able to ameliorate the memory deficits in rats, and the authors suggest that this molecule may compensate central cholinergic defects [58]. Nakahara et al. extended their previous study with rats, and they were examining the effect of cevimeline on memory disorders using a T-maze and radial-arm maze task in experimental amnesia models (bilateral intracerebroventricular injection of cholinotoxine). The repeated administrations of cevimeline for 5 weeks significantly ameliorated impaired performance in a delayed alternation task in the T-maze and acquisition failures in a radial-arm maze task. The authors observed the effect of a single administration of cevimeline, which led to the reduction of the incorrect choices in a radial-arm maze task with 6 h delay time [59]. Fisher et al. found that in cholinotoxine-treated rats, a single dose of cevimeline managed to reverse cognitive impairments in a step-through passive avoidance task. The same was true for reversing reference memory impairments in the Morris water maze test, and the repetitive administrations of cevimeline improved working memory deficits in the same test [60]. The effect of cevimeline on the processes associated with the learning of aged and young rats was studied by Brandeis et al. The Morris water maze task was used for the evaluation of spatial reference memory, while an eight-arm radial maze was used for spatial working memory evaluation. As a result, the age-related cognitive impairments were significantly reduced in both tasks after cevimeline administration [61]. In addition, Vincent et al. evaluated the impact of cevimeline on the learning processes using the Morris water maze task. In this case, model animals were C57BL/10 mice. Learning was significantly improved by ranging dose levels, and U-shaped dose–response dependence was confirmed. The findings show that cevimeline enhanced cognition in mice with a longer duration of action than reported for traditional muscarinic agonists [62]. More specific tests of reference and working memory were used by Dawson et al., including the rat tests of reference memory, a rat response sensitivity test, passive avoidance or conditioned suppression of drinking, and working memory (delayed-matching-to-position) and a mouse tail-flick test. The results of this study suggest that cevimeline can reverse the effects of a scopolamine-induced deficit in these animal tests [68]. The roles of hippocampal M1/M2AChRs in working and reference memory performance of rats were examined in the study of Ohno et al. [64]. The authors applied intrahippocampal injections of selective antagonists (pirenzepine, methoctramine) and studied whether the concurrent injection of cevimeline can reverse the negative effect of these antagonists. It was found that M1 muscarinic receptor agonist cevimeline attenuated increased working memory errors in the presence of both antagonists. The results suggest that processes mediated by M1AChRs in the hippocampus are involved in working memory but not in reference memory [64]. The results of the study of Suzuki et al. suggest that cevimeline is able to ameliorate the disturbance of learning behavior in senescence-accelerated mice. The shortened latency of step-through in this type of mice was prolonged by the administration of cevimeline (3 and 10 mg/kg, p.o.) in a bell-shaped manner. The authors concluded that the central cholinergic system in senescence-accelerated mice may be an appropriate age-dependent model of amnesia for evaluating pharmacological actions of drugs [69]. The positive efficacy of cevimeline on cognition was confirmed in both intravenous and peroral forms of administration in mice by Iga et al. [66]. In the study, the authors evaluated a behavioral memory task, long-term potentiation, and cholinergic modulation of hippocampal rhythmical slow activity. Perorally administered cevimeline in mice resulted in improvement of scopolamine-induced memory deficits at doses of 1.0 mg/kg (passive avoidance task). Intravenously administered cevimeline induced slow rhytmicalhippocampal activity at the same doses of 1.0 mg/kg [66]. O’Neill et al. used a monkey as the only model animal for their experiment. Object working memory was tested in young and aged pretrained monkeys, using a delayed match-to-sample task. Cevimeline was administered by intramuscular injection in doses of 0.1–2.1 mg/kg. The results show that there was an improvement in task performance in both young and aged monkeys. Thus, the findings indicate that cevimeline may represent a low-toxicity treatment of age-related memory disorders [70]. The stimulation of muscarinic receptors (M1AChRs) promotes long-term memory consolidation during and after a fearful experience in mice. Overconsolidation may contribute to the recurrent and intrusive memories of post-traumatic stress disorder. Young et al. illustrated that agonist M1AChRs cevimeline regulates the consolidation of cued fear memory by redundantly activating phospholipase C in the basolateral amygdala [67]. Cevimeline (AF102B) and other structurally related compounds of AF-series developed by Fisher et al. (e.g., AF267B and AF292) act as cognitive enhancers and potential disease modifiers in animal models of AD [71].

The study in human, reported by Nitsch et al., evaluated the effect of cevimeline on the changes in total β-amyloid (Aβ) levels in cerebrospinal fluid (CSF) before and during the treatment. The authors reported data providing evidence of Aβ levels reduction in the AD patients, but they did not measure behavioral or cognitive short-term changes [72]. In an earlier study reported by Fisher et al., clinical trials of cevimeline (AF102B) in AD patients resulted in the dose-dependent improvement in the cognitive subscale of Alzheimer’s disease assessment scale (ADAS-Cog) [73]. It should be noted that xanomeline, the above-mentioned direct mAChR agonist, revealed the affection of cognitive performance at the higher evaluated doses [49]. On the other hand, this may be associated with the adverse effects. Considering the remarkable data in animal studies and a lack of human studies, further research is needed to unambiguously confirm cevimeline’s effect on the improvement of cognitive functions.

### 2.4. Cevimeline and Alzheimer’s Disease

**Figure 4:**
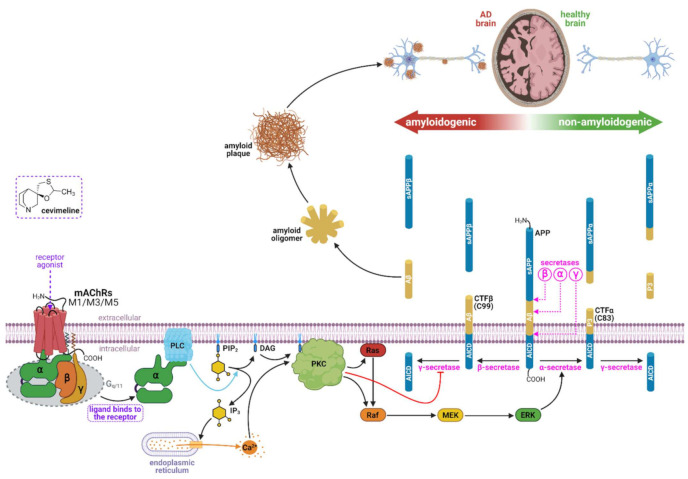
Assumed effect of muscarinic acetylcholine receptor agonists such as cevimeline on the mAChRs in association with AD. The agonist-mediated activation of mAChRs result in increased activity of PKC via same pathway as described in the previous figure. The increased activity of PKC via a cascade of reactions activates ERK, which in turn activates α-secretase. The APP is cleavaged predominantely by α-secretase to produce a non-amyloidogenic P3 peptide. In addition, the activated PKC inhibits γ-secretase activity, leading to a decrease of Aβ in the amyloidogenic pathway. Abbreviations: Aβ, amyloid beta; AICD, APP intracellular domain; APP, amyloid precursor protein; CTFα (C83), C-terminal fragment α; CTFβ (C99), C-terminal fragment β; DAG, diacylglycerol; ERK, extracellular regulated kinase; G_q/11_, G proteins; IP3, inositiol trisphosphate; MEK, mitogen-activated protein kinase kinase; P3, non-amyloidogenic peptidic fragment; PIP2; phosphatidylinositol 4,5-bisphosphate; PLC, phospholipase C; PKC, protein kinase C; Raf, serine/threonine specific protein kinases; Ras, small GTP-binding proteins; sAPP/α/β, secreted APP/α/β. Created with BioRender.com.

It seems that M1AChRs could represent an important target that affects all major features of AD, such as cholinergic deficiency, cognitive dysfunction, Aβ, and tau pathology. Positive effects of muscarinic agonists have been demonstrated in several cell culture studies [74,75,76,77], animal models [78,79], and AD patients [72]. In experiments, cevimeline increased αAPPs, decreased Aβ levels, decreased tau hyperphosphorylation, and blocked Aβ-induced neurotoxicity in vitro (Figure 4). In cognitive tests, it had a positive effect on deficits and showed excellent therapeutic safety, even with various types of administration. 

In vitro experiments on cell lines represent an imaginary beginning to investigate the relationship between cevimeline and a possible AD therapy. Harring et al. investigated the effect of cevimeline on the secretion of amyloid precursor protein (APP) by M1AChRs-transfected PC12 cells. The results indicate that cevimeline might be a molecule suitable for reducing cell-associated APP levels over a prolonged period and thus affect Aβ deposition and possibly delay AD progression [74]. The same authors extended this study (Harring et al.) on the connection with the nerve growth factor (NGF). On NGF-differentiated PC12 cell culture, they promoted APP secretion by administering 50 ng/mL NGF for 3 days. The effects of NGF were reflected by larger reductions in membrane-associated APP levels following muscarinic stimulation [75]. Gurwitz et al. (1995) described an effect of M1-selective muscarinic agonist, which itself caused a minimal neurite outgrowth in M1AChRs-transfected PC12 cells; however, in the presence of NGF, it is able to induce it. The experiment showed that cevimeline might improve the neuronal response to neurotrophic factors and thus contribute to this mechanism in the treatment of AD. Tau pathology is, apart from the presence of Aβ, another characteristic feature of AD [76]. The possibility that tau phosphorylation is controlled by M1AChRs has been investigated by Sadot et al. on M1AChRs-transfected PC12 cells. The stimulation of M1 receptors with cevimeline was found to reduce tau phosphorylation. The effect was time and dose dependent. In addition, a synergistic effect on tau phosphorylation was found when cevimeline was combined with NGF. As the authors conclude, this is the first evidence of a link between the cholinergic signal transduction system and the neuronal cytoskeleton. According to the authors, this can be mediated by regulated phosphorylation of the tau microtubule-associated protein [77]. 

The subsequent in vivo animal experiments have taken previous experiments to the next level of knowledge. Beach et al. extended the results from the in vivo experiments on rabbits, which were administered cevimeline for 5 days, s.c. injections in doses 2 mg/kg/day. They discovered that cevimeline had decreased cerebrospinal fluid Aβ concentrations [78]. Welt et al. used amyloid-β protein precursor transgenic mice as model animals. The results confirmed the effect of modulation of M1AChRs by cevimeline (5 mM applied directly to the hippocampus via retrodialysis) on an Aβ pool in interstitial fluid. These results strengthen the potential of the M1AChRs agonist cevimeline in AD therapy [79].

In 1996, Fisher et al. reported the utilization of cevimeline (AF102B) in human clinical trials performed in Israel, Japan, and the USA. In Israel, a single-blind, placebo-controlled, parallel-group study was evaluated in female and male patients diagnosed with probable AD within 10 weeks. In the study, cevimeline or placebo were orally administered 3 times per day in graduating doses (20 mg, 40 mg, and 60 mg), each dose for 2 weeks. The middle (40 mg) and the highest (60 mg) dose of cevimeline resulted in a significant favorable effect in the ADAS-Cog and word recognition subscale of ADAS. Similarly, the highest dose of cevimeline was effective in patients with moderate to severe dementia. Interestingly, the improvement at the middle and the highest dose of cevimeline was observed by caregivers. The authors claimed that cevimeline was well tolerated, but some adverse effects were observed if compared with the placebo group. However, the most significant adverse effects were of cholinergic nature, such as diaphoresis (20%) and excessive salivation (7%). It has been concluded by the authors that M1 agonists including cevimeline may have a more important and complex role in AD treatment than envisaged previously. Their mode of action might be of unique value in delaying the AD progression [73].

Nitsch et al. explored similar clinical trials in AD patients. They administered cevimeline to 19 AD patients in escalating doses as follows: 3 × 20 mg/day during the first week, 3 × 40 mg/day during the second week, 3 × 60 mg/day during the third week, and 3 × 80 mg/day during the fourth week. Three of the 19 patients with AD died of the causes unrelated to the drug after the study was completed. There were nine men and 10 women with a mean age of 72.2 ± 10.1 (mean ± SD), and the mean duration of their AD was 4.9 ± 2.9 years. The mean information–memory–concentration (IMC) subscale of the Blessed dementia scale score was 19.4 ± 9.8 (Table 1). The most important part of experiment was a measuring of total Aβ levels in cerebrospinal fluid (CSF) before and during treatment. Total levels of Aβ in CSF decreased by 22% in 14 patients, increased in three patients, and were uncharged in two patients. These results revealed a statistically significant overall decrease in the group. The authors concluded that there was evidence that the activation of M1AChRs reduced Aβ levels in the CSF of AD and may have long-term therapeutic benefits by lowering amyloid in AD [72].

Despite the current approach to the treatment of AD focused on the application of acetylcholinesterase (AChE) inhibitors, agonists of mAChRs may represent an alternative therapy to increase the neuronal stimulation and improve cognitive functions. Clinical trials of cevimeline and xanomeline revealed that mAChR agonists were feasible drugs with the potential to improve the cognitive abilities of AD patients. Same as the AChE inhibitors, these mAChR agonists also displayed side effects, especially of gastrointestinal nature. However, cevimeline along with other mAChR agonists still attracts attention and discussion as a potential drug for developing new therapies for cognitive enhancement [80]. The beneficial effects of cevimeline in AD patients can overcome its adverse effects to allow a more comfortable life for the patients and their families, but further clinical studies are needed to elucidate cevimeline’s therapeutic effect against AD.

## 3. Evoxac^®^ (Cevimeline Hydrochloride) in the Treatment of Sjögren’s Syndrome

Increased salivation and tearing in animal and human trials with cevimeline became an inspiration for the treatment of xerostomia (subjective sensation of dry mouth) and keratoconjunctivitis sicca (syndrome of dry eye), which are the most common symptoms in patients with Sjögren’s syndrome. The clinical studies with cevimeline turned their attention toward the patients with Sjögren’s syndrome for the evaluation of safety and efficiency. Petrone et al. performed double-blind, randomized, placebo-controlled study in patients with the syndrome. In the study, patients received either placebo, 15 mg of cevimeline, or 30 mg of cevimeline, each 3 times per day for 12 weeks. The treatment with the highest dose (30 mg) resulted in the significant improvement of salivation and lachrymation in the patients. The authors reported that subjective symptoms in this group (30 mg), such as dry eyes, dry mouth, and overall dryness were improved. The group of patients with the lower dosage of cevimeline (15 mg) also relieved some symptoms. Both dosages (15 mg and 30 mg) were well tolerated by the patients. The most common adverse effects in the study include increasing sweating, headache, abdominal pain, and nausea [81]. In the same year, Fife et al. performed a double-blind, randomized, placebo-controlled study of cevimeline’s safety and efficiency in the treatment of xerostomia in Sjögren’s syndrome patients. In the study, participants received either placebo, 30 mg of cevimeline or 60 mg of cevimeline, each 3 times per day for 6 weeks. The study, completed on 61 participants, revealed that both dosages provided symptomatic improvement. However, the lower dosage of cevimeline (30 mg) was well tolerated, whereas the highest dosage of cevimeline (60 mg) was associated with adverse effects, commonly of a gastrointestinal nature. The most common adverse effects usually reported in the group with the highest dosage (60 mg) involve: increasing sweating, nausea, headache, rigors, diarrhea, dyspepsia, dizziness, and abdominal pain. Nevertheless, the authors concluded that therapy with cevimeline at the dose of 30 mg 3 times per day provided significant improvement of xerostomia, and this dosage seems to be well tolerated [82].

Evoxac^®^, the brand name of cevimeline hydrochloride, was approved in January 2000 by the Food and Drug Administration (FDA) in the USA, as a drug for the treatment of Sjögren’s syndrome. Evoxac^®^ is available as white capsules containing 30 mg of cevimeline hydrochloride with recommended dosage of one capsule 3 times per day. The package leaflet of Evoxac^®^ involves the information of adverse reactions from the worldwide clinical trials in 1777 patients, including Sjögren’s patients and patients with other conditions. The adverse events observed in the clinical trials of cevimeline in Sjögren’s syndrome patients associated with muscarinic agonism include excessive sweating (18.7%), nausea (13.8%), rhinitis (11.2%), diarrhea (10.3%), excessive salivation (2.2%), and other [83]. In 2021, Fox et al. reported that the only approved oral secretagogue agents by the FDA were cevimeline and pilocarpine, and according to the European Medicines Agency (EMA), it was pilocarpine [84]. These agents act by stimulating M1 and M3 receptors on the salivary glands, resulting in the increased function of secretion [85]. Cevimeline and pilocarpine were investigated by Farag et al. in patients with hyposalivation to compare their effectiveness and frequency of adverse effects. The authors reviewed a total of 110 patients’ charts and concluded that the effectiveness of both agents was comparable; however, pilocarpine seemed to be more linked with the reporting of side effects [86]. To the best of our knowledge, there is only one example of cevimeline overdose reported to date. A 47-year-old female intentionally ingested 20 capsules of cevimeline (30 mg), 2 tablets of lorazepam (0.5 mg) earlier in the day, and drank some alcohol. The sose of cevimeline was reported to reach approximately 10 mg/kg and the symptoms of moderate muscarinic toxicity were observed. In the patient, extremely diaphoresis and nausea were reported with several episodes of emesis. At the emergency department, the electrocardiogram monitoring of the patient demonstrated that sinus rhythm, axis, and QRS width were at regular levels. To get rid of nausea, ondansetron was administered to the patient, as well as activated charcoal (50 g). Further monitoring revealed that the patient remained normotensive and without dysrhythmia. The patient was fully recovered during the hospitalization and did not exhibit any delayed impairment of ingestion [19].

Despite the above-mentioned cevimeline’s adverse effects, its application in the treatment of Sjögren’s syndrome provides an improvement of symptoms, such as dry mouth and dry eye. Moreover, a dosage of cevimeline 3 × 30 mg per day is approved by the FDA and mediates observable benefits only with reduced side effects. Extensive use of cevimeline in the treatment of Sjögren’s syndrome provided valuable information about the pharmaceutical profile of the agent. Although cevimeline has been used as the drug for decades, many questions about its extended application, such as its use in the AD therapy or in the improvement of cognitive functions, still remain unanswered. Its potential was suggested in previous studies, but further studies are needed. This request is still present in recently reported literature. Even if cevimeline’s benefits will not overcome its adverse effects, its structure can be used as a template for a novel group of potential therapeutics.

## 4. Methods

**Figure 5:**
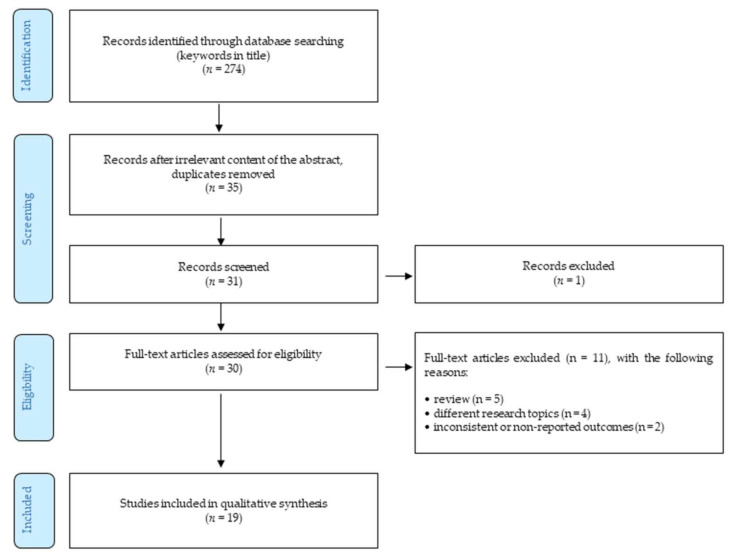
Selection workflow.

The authors performed a systematic literature review of available studies on the research describing cevimeline in association with neurodegenerative disease. The methodology follows the Preferred Reporting Items for Systematic Reviews and Meta-Analysis (PRISMA) guidelines (Figure 5). The end of the search period is limited by 31 March 2021. The research studies were selected based on research topics, such as “Cevimeline, Evoxac, AF102B, SNI2011, cognition, memory, neurodegeneration, Alzheimer disease” found in the world’s acknowledged databases Web of Science, PubMed, Springer, and Scopus. The terms used were searched using AND to combine the keywords listed and using OR to remove search duplication where possible. With the aim of deeper insight, a backward search was also performed; i.e., the authors evaluated the references of the detected studies to find other relevant research studies that authors might have missed during their search. Moreover, the identification of unpublished (gray) literature was conducted using a Google search. Furthermore, an independent quality assessment of selected studies was performed. These basic quality criteria were selected by authors using the Health Evidence Quality Assessment Tool for review articles. The primary outcome of this review was to explore if there is any possibility of using cevimeline into new neurodegenerative disease treatment or prevention.

Altogether, 274 studies were identified in all these databases. The following selection consisted of removing duplicates and studies unrelated to the research topics (considering title/abstract) and provided 35 English-written studies. Of these, the authors selected 30 articles relevant for the research topic. These studies were fully investigated and considered against the following inclusion and exclusion criteria:

- Only peer-reviewed English-written full-text journal articles were involved.
- The time of publishing the article was limited by 31 March 2020.

The exclusion criteria were as follows:

- Reviews.
- The articles focusing on different research topics (such as xerostomia, dry mouth, etc.).
- Outcomes were not reported or were inconsistent.

Considering the above-described criteria, 19 articles were eventually included in the final analysis.

## 5. Conclusions

Cevimeline is a parasympathomimetic and muscarinic agonist with special effects on M1 and M3 receptors. It is usually indicated for the treatment of the symptoms of dry mouth in patients with Sjögren’s syndrome. However, this systematic literature review indicated other possibilities for its use in medicine. The results of the detected studies indicate that cevimeline has shown an improvement in experimentally induced cognitive deficits in animal models. Furthermore, it has demonstrated to positively influence tau pathology and reduce the levels of amyloid-β (Aβ) peptide in the cerebral spinal fluid of Alzheimer’s patients. Although this drug has not been approved by the FDA for its use among AD patients, and there is a lack of clinical studies confirming and extending this finding, cevimeline might represent a potential remedy in the treatment of AD. 

## Figures and Tables

**Table 1:** Clinical trials of cevimeline, hydrocychloroquine, and physostigmine in AD patients as reported by Nitsch et al. [72].

	Total Number of AD Patients in the Trials (Female/Male)	Age [Years] (Mean ± SD)	Illness Duration [Years] (Mean ± SD)	IMC Score * (Mean ± SD)	Baseline CSF Aβ_total_ (Mean ± SD)	Treatment CSF Aβ_total_ (Mean ± SD)
Cevimeline	19 (10/9)	72.2 ± 10.1	4.9 ± 2.9	19.4 ± 9.8	22.8 ± 7.5	19.9 ± 7.9
Hydroxychloroquine	10 (3/7)	68.3 ± 6.9	3.8 ± 1.9	19.5 ± 13.1	23.5 ± 10.1	24.0 ± 8.1
Physostigmine	9 (6/3)	67.7 ± 9.3	3.8 ± 1.5	13.2 ± 8.9	21.2 ± 7.9	21.0 ± 6.1

Abbreviations: IMC, information–memory–concentration; SD, standard deviation. * The score on the IMC subtest of the Blessed dementia scale.
